# Retraction Note: Effect of neoadjuvant chemotherapy combined with arterial chemoembolization on short-term clinical outcome of locally advanced gastric cancer

**DOI:** 10.1186/s12885-025-15033-8

**Published:** 2025-10-28

**Authors:** Jianguo Yang, Juncai Li, Qican Deng, Zhenzhou Chen, Kuan He, Yajun Chen, Zhongxue Fu

**Affiliations:** 1 Department of Gastrointestinal surgery, The First Affiliated Hospital of Chongqing Medical University, Chongqing, China; 2 Department of Thoracic Surgery, Yubei District people’s Hospital of Chongqing, Chongqing, China

**Retraction Note: BMC Cancer 23, 246 (2023)**

**https://doi.org/10.1186/s12885-023-10712-w**

The authors have retracted this article because after its publication, they noticed significant errors in the statistical analysis and data processing. The subgroup ypT0 was erroneously classified as pathological complete response (pCR). However, some patients who were ypT0 but lymph node-positive were incorrectly counted as pCR patients.

Yajun Chen has not responded to any correspondence from the editor/publisher about this retraction. All other authors agree to this retraction.

